# ADAMTS Proteases: Importance in Animal Reproduction

**DOI:** 10.3390/genes14061181

**Published:** 2023-05-29

**Authors:** Pamela Hernández-Delgado, Monserrath Felix-Portillo, José A. Martínez-Quintana

**Affiliations:** Facultad de Zootecnia y Ecología, Universidad Autónoma de Chihuahua, Chihuahua 31453, Mexico; p277761@uach.mx (P.H.-D.); monserrath.felix@uach.mx (M.F.-P.)

**Keywords:** animal reproduction, ADAMTS, extracellular matrix, genetic improvement

## Abstract

Many reproductive physiological processes, such as folliculogenesis, ovulation, implantation, and fertilization, require the synthesis, remodeling, and degradation of the extracellular matrix (ECM). The *ADAMTS* (A Disintegrin and Metalloproteinase with Thrombospondin Motifs) family genes code for key metalloproteinases in the remodeling process of different ECM. Several genes of this family encode for proteins with important functions in reproductive processes; in particular, *ADAMTS1*, 4, 5 and 9 are genes that are differentially expressed in cell types and the physiological stages of reproductive tissues. ADAMTS enzymes degrade proteoglycans in the ECM of the follicles so that the oocytes can be released and regulate follicle development during folliculogenesis, favoring the action of essential growth factors, such as FGF-2, FGF-7 and GDF-9. The transcriptional regulation of *ADAMTS1* and 9 in preovulatory follicles occurs because of the gonadotropin surge in preovulatory follicles, via the progesterone/progesterone receptor complex. In addition, in the case of ADAMTS1, pathways involving protein kinase A (PKA), extracellular signal regulated protein kinase (ERK1/2) and the epidermal growth factor receptor (EGFR) might contribute to ECM regulation. Different *Omic* studies indicate the importance of genes of the *ADAMTS* family from a reproductive aspect. *ADAMTS* genes could serve as biomarkers for genetic improvement and contribute to enhance fertility and animal reproduction; however, more research related to these genes, the synthesis of proteins encoded by these genes, and regulation in farm animals is needed.

## 1. Introduction

The enormous expansion in the production of food from livestock during the last 70 years has been possible, in great part, through genetic improvement [1]. Reproduction is one of the most relevant aspects in animal production, because of relevancy to productivity and sustainability of the different livestock systems [2]; however, these traits, as well as other economically important traits, are quantitative, multifactorial and have low heritability. The development of molecular and genomic technologies has contributed to the identification of major genes and genetic markers associated with phenotypes, leading to accelerated genetic improvement [3]. Nevertheless, it is important to elucidate how genes that encode for the proteins related to such traits are regulated and how the genetic variants affect their expression [4].

Extracellular matrix (ECM) remodeling is an important physiological process related to reproductive capacity in males and females. This restructuring of the ECM has been associated with the reproductive processes of folliculogenesis, ovulation, implantation and placentation [5,6] in females and in processes that induce correct testicular development, as well as the ongoing production and maturation of millions of spermatozoa in males [6,7]. In addition, the degradation of the ECM in the zona pellucida is an important physiological process for oocyte fertilization [8].

Different matrix metalloproteinases function in the degradation of extracellular matrices [9]. The metalloproteinases superfamily is composed of ADAM proteases (A Disintegrin-like and Metalloproteinase), MMPs (Matrix Metalloproteinases), astacins (BMP/tolloid proteases and meprins in mammals) and ADAMTS (A Disintegrin-like and Metalloproteinase Domain with Thrombospondin type 1 repeat) [10]. Both MMPs and ADAMTS are secretory proteins. ADAMTS are key proteases in the degradation of the ECM, particularly cleaving large proteoglycans, whereas MMPs recognize short peptides as substrates and thus have a wider range of protein targets and a role in many different physiological processes [11,12]. In contrast, the ADAM proteases are integral membrane enzymes that mainly cleave ectodomains of different secretory proteins [13].

In addition to the crucial role of the ADAMTS proteases in the ECM remodeling of the development of reproductive organs and processes, Etacin-related proteins also have an important function, activating growth factors necessary for the assembly of the ECM [14,15,16]. Thus, the study of these genes can contribute to the full understanding and improvement of the reproductive processes and can potentially impact the sustainability of animal production. In this article, we describe the general structure of the *ADAMTS* genes, the importance of ADAMTS proteases in reproduction biology and the biosynthesis regulation of these proteins, as determined in reproductive genomics studies.

## 2. Structural Features of ADAMTS Genes

ADAMTS proteinases are multidomain enzymes with highly conserved structures [17]. *ADAMTS1* was the first gene of this family to be described in mice [18], and later, other genes were identified in other species. In mammalian genomes, 19 *ADAMTS* genes have been identified and named *ADAMTS1* to *ADAMTS20*. It was later discovered that *ADAMTS5* and *ADAMTS11* are the same gene, and *ADAMTS11* is no longer used [19,20]. The expansion in the number of *ADAMTS* genes in mammals seems to have occurred due to gene duplication, thus generating sub-functionalization or neo-functionalization regarding the physiological processes in which they participate [13]. Rose et al. [21], in their excellent review, explain that *Gon-1* is the only *ADAMTS* orthologous gene in *Caenorhabditis elegans*, and it has similarity to *ADAMTS9* and *ADAMTS20* in humans. The six ADAMTS proteases in the ascidian *Ciona intestinalis* represent the central evolutionary clades in chordates from which gene expansion into vertebrates occurs [22], along with the evolution of the ECM. Phylogenetic analysis clearly suggests the gene duplication of the *ADAMTS* genes [21].

**Figure 1:**
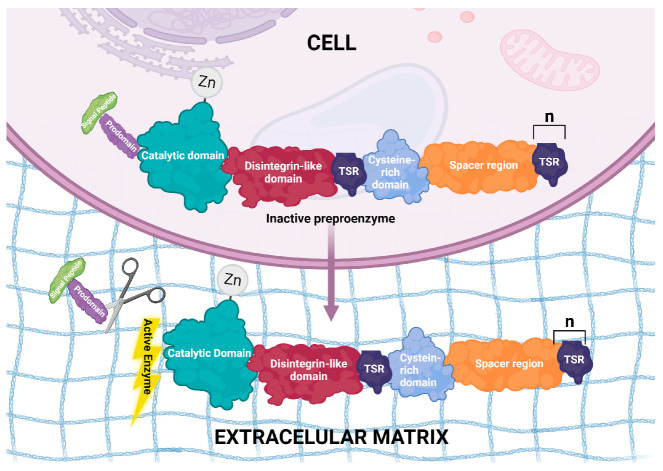
Illustration of ADAMTS protein structure and localization. Cleavage can occur in trans-Golgi, on the cellular surface or extracellularly.

The signature domains of the ADAMTS proteins are: a signal peptide, necessary for protein trafficking and secretion, and an inhibitory prodomain that must be cleaved from the ADAMTS zymogens to render them catalytically active. Such cleavage occurs in Golgi, the cell surface or extracellularly. The size of this prodomain comprises about 200 residues in all ADAMTS proteases, with the exception of ADAMTS13, which has a short prodomain that does not need to be removed for the protease to be active [23]. Interestingly, the removal of the ADAMTS9 prodomain reduces the protease catalytic activity upon versican, its substrate; a disintegrin-like domain; a thrombospondin type 1 repeat (TSR-1) motif; and a cysteine-rich domain followed by a spacer region. The TSR-1 domains and the spacer domain appear to be involved in ECM anchoring. The description of the organization of these proteins is based on the structure of ADAMTS4, the other members of the family vary mainly at the C-terminus, with either, more or fewer repeats of the TSR-1 motifs [24]. Figure 1 illustrates the structure and localization of these proteins.

The *ADAMTS* genes are located on different chromosomes that vary depending on the species. These genes code for proteins with a theoretical weight ranging between 70.9 and 225.64 kDa, with 662 amino acid residues in species such as the rooster (*Gallus gallus*) or as many as 2028 in species such as the pig (*Sus scrofa*). Table 1 details the structural characteristics of these genes and their protein products in farm animals, comparing these with the mouse sequences. It is interesting to note that in sheep (*Ovis aries*), the *ADAMTS1*, 4 and 5 genes are found on the same chromosome (Table 1). These genes are thought to encode for proteins involved in the regulation of reproductive functions. In the case of cattle, humans and mice, genes 1 and 5 are located in the same chromosome, as reported by Dubail and Apte [13].

According to different studies, the ADAMTS family proteins have functions in tissue remodeling during the development of organs and reproductive processes [6]. The reproductive function of proteins encoded by these genes has been extensively studied in mice, while studies in farm animals are scarce; however, as shown in Table 2, there is a high ADAMTS protein similarity among species.

## 3. ADAMTS and Fertility in Females

### 3.1. Folliculogenesis

The process of development and maturation of follicles, termed folliculogenesis, is necessary for ovulation to occur [25]. The *ADAMTS* family genes seem to be related to this process, inferred by the relative abundance of mRNA transcripts in the follicles and corpus lutea of several mammalian species. *ADAMTS1* have been reported to be expressed in granulosa cells in cows [26], horses [27] and pigs [28].

According to Brown et al. [29], ADAMTS1 functions are necessary for the structural changes of the ECM to occur during follicular development. The proteoglycans present in the ECM can inhibit the action of certain growth factors, such as FGF-2, FGF-7 and GDF-9 [30], which are essential for various exquisite reproductive processes to occur. For example, FGF-2 stimulates angiogenesis and granulosa cell proliferation and function in cattle [31] and buffalo [32]. FGF-2 also stimulates the initiation and development of follicular growth in sheep and goats [33,34].

Thus, the functions of ADAMTS1 and 4 are thought to enhance these processes by controlling the amount and location of various proteoglycans [35]. Shozu et al. [36] inactivated the *ADAMTS1* gene and reported that the absence of ADAMTS1 led to follicular atresia. Versican is an abundant ECM proteoglycan that is hormonally regulated by the ovary. Versican abundance varies throughout the several stages of follicular growth but particularly during ovulation in rodents [37]. The presence of versican in bovine and porcine follicular basement membrane [38,39], suggests that ADAMTS1 may also regulate the development of follicles during folliculogenesis.

### 3.2. Ovulation

The preovulatory surge of gonadotropins induces a series of biochemical processes within the dominant follicle that culminate in ovulation and, subsequently, in the formation of the corpus luteum. Ovulation is associated with the degradation of the follicular basement membrane and the fragmentation of the ECM at the apex of the follicle wall, resulting in the release of the oocyte [40]. Metalloproteinases enzymes are responsible for the degradation of the follicular ECM during ovulation [9]. ADAMTS1 degrades versican, aggrecan and brevican, proteoglycans present in the ECM of the follicle. Such degradation of the follicular wall allows oocyte release [41,42]. Indeed, ADAMTS1 was reported to have a fundamental function in ovulation, as reported by Mittaz et al. [43], based on results from a study with female mice lacking the ADAMTS1. In this study, exon 2 was deleted to disrupt the *ADAMTS1* gene and a selectable marker gene was inserted in intron 1. The modified *ADAMTS1* allele was functionally null. The authors reported that these females were subfertile due to impaired ovulation, resulting in the mature oocytes not being released from the follicles, as would typically occur during ovulation. Likewise, Brown et al. [44] reported that the ovulation rate was 77% less in female mice lacking the ADAMTS1 enzyme, compared to the wild-type animals. The finding was explained by the lack of versican degradation during the matrix expansion of the cumulus–oocyte complex.

*ADAMTS1* and *ADAMTS9* mRNA abundance was increased in the granulosa [45] and theca cells [46] of periovulatory follicles compared with the preovulatory follicles of cattle. This was also observed in vitro in response to LH stimulation in theca cells and to LH or FSH stimulation in granulosa cells [47], indicating the importance of the ADAMTS proteins in ovulation, as these enzymes are necessary for the disruption of the ECM integrity of the follicles and the subsequent release of the oocytes [46]. In mice, there is a marked increase in the mRNA expression of the *ADAMTS4* gene in granulosa cells before ovulation and in different cell types after ovulation, including the site where follicle wall degradation occurs [16]; however, when studying expression in cattle, Madan et al. [26] did not observe changes in the *ADAMTS4* mRNA transcript abundance in granulosa or theca cells in periovulatory compared to preovulatory follicles. On the other hand, in a transcriptome sequencing study, *ADAMTS4* was one of the genes with the highest relative expression, based on mRNA transcript abundance in the ovarian tissue of prolific compared to non-prolific sheep [48].

**Figure 2:**
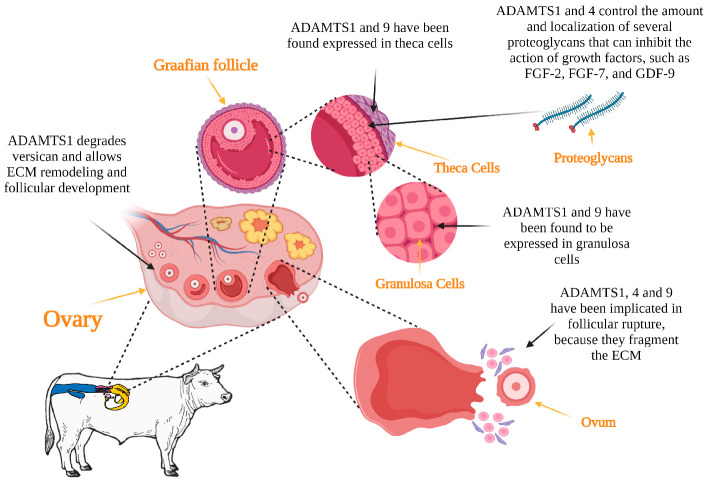
ADAMTS genes are involved in folliculogenesis and ovulation. The specific gene, site of expression and function is shown.

Hu et al. [49] reported that SNP-type polymorphisms in *ADAMTS1* are related to litter size in goats; therefore, they could be used as molecular markers for the selection of litter size. *ADAMTS1*, 4 and 9 are considered to be of great importance for animal production, where reproductive prolificacy is a determinant for sustainability. Figure 2 depicts the possible functions of ADAMTS proteins in folliculogenesis and ovulation in livestock.

### 3.3. Implantation, Placentation and Parturition

Similar to the constant remodeling of the ECM required for the cyclical transformations of the ovary, the uterus also undergoes the cyclic development and remodeling of the endometrial tissue matrix. This remodeling is necessary for implantation and placentation [50]. Implantation is a critical process for the establishment of pregnancy and begins with essential signaling from the blastocyst to the endometrium, which must be prepared to respond [51,52]. The attachment of the blastocyst to the uterus and subsequent trophoblast cell invasion occurs through ECM remodeling [53]. Uterine tissue remodeling is also required for placental cotyledon formation and angiogenesis near trophoblast tissue in sheep, as well as a decrease in endometrial thickness during implantation [54,55]. Shindo et al. [56] reported that non-ADAMTS1 female mice had thicker uteri compared to wildtype females, and such uterine thickening negatively affected fertilization. In addition, *ADAMTS1* is expressed in the endometrium during the estrous cycle of cattle and at the time of uterine remodeling for implantation and placental development. Mishra et al. [57] reported that *ADAMTS1* was more abundant in the last stage of gestation in the cotyledonary tissues of the placenta. This could be because the remodeling of the endometrial ECM leads to the modification of the composition in response to implantation signals. *ADAMTS5* and *ADAMTS6* genes are expressed in the mouse placenta and *ADAMTS5* is expressed in the 7-day embryo but not in the later stages of development [17]. The remodeling of the uterine ECM, therefore, was proposed to be essential for embryonic/fetal development.

**Figure 3:**
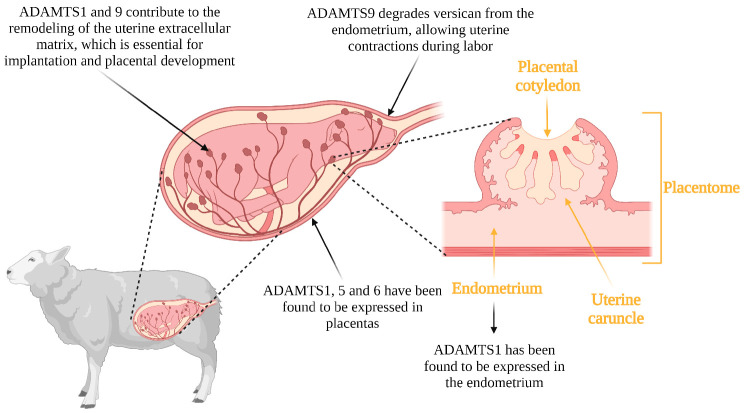
ADAMTS genes are involved in implantation, placentation and parturition. The specific gene and its site of expression and function is shown.

Another member of this family of metalloproteinases, ADAMTS9, has been reported to contribute to remodeling in the uterus at the time of parturition. The extracellular matrix undergoes remodeling during late gestation to allow smooth muscle cells to connect to each other and effect uterine contractions at the time of parturition [58]. ADAMTS9 is present in all reproductive states and contributes to uterine tissue remodeling. The accumulation of versican from the extracellular matrix in the uterus leads to abnormal contractions. Mead et al. [59] reported that there are abnormally large concentrations of versican in mice that do not produce ADAMTS9, leading to abnormal parturition processes. This abnormality was due to a reduction in focal adhesions between cells that interact with one another to generate uterine contractions. Thus, ADAMTS9 contributes to the remodeling of the uterine extracellular matrix through the degradation of versican, and its null or poor functionality disrupts parturition processes. The possible functions of the ADAMTS proteases in implantation, placentation and parturition in livestock is illustrated in Figure 3.

## 4. ADAMTS and Fertility in Males

### 4.1. Testicular Development

The expression of *ADAMTS* family genes, in addition to being related to fertility in females, has also been linked to reproductive capacity in males, specifically to testicular development [60]. The testicles develop in the abdomen during the embryonic and fetal stages. Subsequently, the testes pass through the inguinal canal into the scrotum [7], with this process requiring the tissue remodeling of the ECM [6]; however, regarding this process, the literature is limited to only a few studies of rodents. Jacobi et al. [61] reported there was expression of *ADAMTS16* in the testes of mouse embryos. Likewise, alterations in *ADAMTS16* led to cryptorchidism and infertility in male rats [60,62]. Nevertheless, as reported by Livermore et al. [63], the silencing of *ADAMTS16* via CRISPR/Cas9 gene editing led to the production of mice that were fertile, although with smaller testicles. On the other hand, Carré et al. [64] reported that there was a greater abundance of *ADAMTS12* expressed in developing testicular cords at the time of sex determination in poultry. Only these two genes of the *ADAMTS* family (12 and 16) have been linked to testicular development, and more research is needed to elucidate ADAMTS functions during the development of gonads in different species.

### 4.2. Spermatogenesis

Spermatogenesis is the process through which germ cells multiply and differentiate to produce sperm in the seminiferous tubules [65]. The presence of *ADAMTS10* expression in the testis, epididymis and ejaculated spermatozoa of Asian buffalo (*Bubalus bubalis*) [66] suggests a possible function in the sperm maturation process [67]. ADAMTS2 has also been linked to sperm maturation in mice, as shown by Li et al. [68]. In transgenic mice homozygous for the inactive alleles of *ADAMTS2*, there was less sperm maturation and activity compared to those in the control group; however, more research is needed to determine the functions of these proteins in sperm maturation.

In bulls with besnoitiosis compared to healthy bulls, *ADAMTS1* mRNA abundance is lower in scrotal skin, the pampiniform plexus and testicular parenchyma [69]. This disease causes the fibrosis and thickening of the skin of the scrotum, which leads to failure in thermoregulation and to the inhibition of spermatogenesis, ultimately causing infertility [70].

Wu et al. [71] performed a transcriptomic analysis of yak and cattleyak testes to investigate the genetic causes of hybrid animal sterility, and several *ADAMTS* genes were differentially expressed. *ADAMTS1*, 10, 12, 3, 5 and 14 were upregulated, whereas *ADAMTS16*, 20, 6 and 18 were downregulated; thus, these proteins could be involved in cattleyak sterility. However, how these proteins are associated with hybrid animal sterility is still unclear.

### 4.3. Fertilization

ADAMTS is apparently involved in sperm and egg fertilization processes. In a study conducted by Dun et al. [8] in mice, *ADAMTS10* was expressed during the late stages of spermatogenesis, and the protein was incorporated into the acrosome of developing spermatids. ADAMTS10 presence in the acrosome is thought to function by inducing sperm adhesion to the zona pellucida. The zona pellucida is an ECM that surrounds the oocytes and must be crossed by spermatozoa to penetrate the oocyte and carry out fertilization. ADAMTS10 has important functions in this process by acting in the degradation of the zona pellucida [72]; however, further research in farm animals is needed to understand the role of ADAMTS10 in fertilization.

## 5. Regulation of *ADAMTS* Genes Involved in Reproduction

The ADAMTS proteases family is formed of 19 members that have actions on a huge range of substrates and in different tissues. In an excellent review about the *ADAMTS* regulation [21], the authors highlight the importance of the regulation of these metalloproteinases in different human pathologies, such as arthritis, where pro-inflammatory molecules promote the transcriptional overexpression of the *ADAMTS* genes.

The transcription of the *ADAMTS* genes in reproduction-related processes is hormonally regulated. The gonadotropin surge that induces ovulation leads to a series of drastic changes in the follicles. *ADAMTS1*, 2, 7 and 9 are regulated in vivo by the LH/FSH surge in the ovarian theca cells of cattle, in a time-dependent manner, whereas results from in vitro studies confirmed the effect of LH on *ADAMTS1* and 9 but not on *ADAMTS2*; however, using the same in vitro cell model, the treatment with mifepristone, a progesterone receptor inhibitor, blocked the up-regulatory effect of LH in periovulatory theca cells [46]. These results confirm that the gonadotropin surge regulates the *ADAMTS1* and 9 transcription through the progesterone/progesterone receptor (PGR) complex, as was also reported for *ADAMTS1* in follicle granulosa cells of cattle [47], horses [27] and pigs [28].

In vitro studies with cattle granulosa cells stimulated by forskolin showed a greater abundance of *ADAMTS1*, thus providing a good model to study the regulation of this gene. Using different fragments of the *ADAMTS1* promoter region and luciferase as a reporter gene, Sayasith et al. [73] determined that the deletion between −330 and −177 led to a marked reduction in both basal promoter activity and forskolin-stimulated promoter activity, with the Ebox elements located in this region appearing to play a key role in the regulation of *ADAMTS1* transcription. Investigating the possible pathway for transcription regulation, it was determined that protein kinase A (PKA), extracellular signal-regulated protein kinase (ERK1/2), epidermal growth factor receptor (EGFR) and progesterone receptors (PGR) have a potential role in the activation of *ADAMTS* promoter in cattle.

Little is known about the regulation of *ADAMTS* genes related to reproductive biology in farm animals; therefore, further research should be conducted. In addition, from what is described in this review article, it is important to consider that these genes have a large number of post-transcriptional and post-translational modifications, some of which have already been described in humans and mice and excellently compiled by Rose et al. [21].

## 6. *ADAMTS* in *Omics* Studies

The genomics era has evolved throughout the past two decades. Genomics and transcriptomic studies have been conducted in different animal species to determine what genes are present in these genomes, to identify genetic variations and their possible association with traits of interest and to determine differences in transcription levels in different cell types, environmental conditions, physiological stages, etc.

Wu et al. [71] performed a transcriptomic analysis of yak and cattleyak testes to investigate the genetic causes of hybrid sterility. The mRNA abundance in several *ADAMTS* family genes was different: *ADAMTS1*, 10, 12, 3 and 5 were over-expressed and *ADAMTS16*, 20, 6 and 18 were under-expressed in cattleyaks in comparison to yaks. In transcriptomes analyzed in uterine tissue at day 6 post-insemination, genes related to ECM remodeling had differential mRNA transcript abundance in pregnant and non-pregnant cows, 30 days post-insemination. *ADAMTS1* was among these genes, indicating the importance of this protein in the uterine receptivity for the embryo to ensure the sustaining of pregnancy [74]. In an in vitro study, *ADAMTS20* was one of the 20 over-expressed genes in the granulosa cells of cattle in response to severe heat stress [75]. In sheep, where prolificacy is a very important aspect, *ADAMTS4* and *ADAMTS14* were overexpressed in the whole ovarian transcriptomes of Pelibuey ewes with greater prolificacy compared with ewes with less prolificacy [48]. Furthermore, Gootwine [76] suggests further investigation on the possible relationship between ADAMTS9 and uterine capacity in sheep, considering the relative abundances of these gene-transcripts in different genomic studies in pigs and sheep. Transcriptomic studies and genome scans can be conducted to identify genes for the further analysis of functionality and regulation.

## 7. Conclusions

The results of many studies indicate the *ADAMTS* family genes are involved in the reproductive biology of both males and females in farm animals; however, further research is required to elucidate the specific functions of the products of these genes and the regulation of their expression and effects of environmental conditions, as well as the genotype:environment:phenotype integration. With such integrative understanding, more strategies can emerge that will allow genetic improvement and gradually enhance the sustainability of animal production systems.

## Figures and Tables

**Table 1:** Characteristics of *ADAMTS* genes in livestock and mice.

Gene	Species	Chr	Exon No	Size (bp)	AA No	Gene	Species	Chr	Exon No	Size (bp)	AA No
ADAMTS1	*Mus musculus*	16	9	9287	968	ADAMTS9	*M. musculus*	6	40	171,752	1931
	*Bos taurus*	1	9	8698	970		*B. taurus*	22	41	163,000	1937
	*Ovis aries*	1	9	9452	968		*O. aries*	19	41	162,411	1935
	*Equus caballus*	26	9	9002	949		*E. caballus*	16	40	166,329	1936
	*Sus scrofa*	13	9	8783	947		*S. scrofa*	13	40	175,486	2028
	*Gallus gallus*	1	9	6915	923		*G. gallus*	12	41	67,065	1928
ADAMTS2	*M. musculus*	11	22	205,489	1213	ADAMTS10	*M. musculus*	17	26	30,137	1104
	*B. taurus*	7	22	245,248	1205		*B. taurus*	7	26	21,940	1103
	*O. aries*	5	23	255,023	1206		*O. aries*	5	27	21,873	1103
	*E. caballus*	14	23	215,470	1211		*E. caballus*	7	27	21,061	1191
	*S. scrofa*	2	23	223,979	1208		*S. scrofa*	2	24	16,972	1103
	*G. gallus*	13	27	236,034	1187		*G. gallus*	28	25	47,963	1109
ADAMTS4	*M. musculus*	1	9	12,139	833	ADAMTS12	*M. musculus*	15	24	284,541	1600
	*B. taurus*	3	9	8057	839		*B. taurus*	20	25	394,653	1607
	*O. aries*	1	9	8091	872		*O. aries*	16	24	402,782	1605
	*E. caballus*	5	9	7009	837		*E. caballus*	21	24	314,874	1598
	*S. scrofa*	4	9	8885	872		*S. scrofa*	16	24	373,778	1599
	*G. gallus*	25	9	7976	662		*G. gallus*	Z	23	170,964	1580
ADAMTS5	*M. musculus*	16	9	42,969	930	ADAMTS16	*M. musculus*	13	24	114,040	1222
	*B. taurus*	1	9	50,445	934		*B. taurus*	20	23	193,779	1246
	*O. aries*	1	8	57,118	934		*O. aries*	16	23	183,599	1226
	*E. caballus*	26	9	49,839	929		*E. caballus*	21	23	154,339	1249
	*S. scrofa*	13	10	60,092	929		*S. scrofa*	16	24	164,333	1238
	*G. gallus*	1	8	46,807	877		*G. gallus*	-	-	-	-
ADAMTS6	*M. musculus*	13	28	210,178	1117	ADAMTS18	*M. musculus*	8	24	152,221	1219
	*B. taurus*	20	26	286,175	1117		*B. taurus*	18	24	145,453	1223
	*O. aries*	16	28	289,952	1117		*O. aries*	14	21	147,254	1050
	*E. caballus*	21	25	358,367	1117		*E. caballus*	3	23	125,706	1199
	*S. scrofa*	16	24	308,302	1117		*S. scrofa*	6	23	164,005	1224
	*G. gallus*	Z	26	159,448	1119		*G. gallus*	11	23	69,793	1228

Chr = chromosome location, AA = amino acids. Data from NCBI (https://www.ncbi.nlm.nih.gov/) accessed on 10 March 2023.

**Table 2:** Identity and similarity percentage of ADAMTS proteins between *M. musculus* and farm animals.

Species	ADAMTS																			
	1		2		4		5		6		9		10		12		16		18	
	I	S	I	S	I	S	I	S	I	S	I	S	I	S	I	S	I	S	I	S
*B. taurus*	81	87	89	93	88	92	88	90	96	97	88	93	95	96	78	85	78	85	86	91
*O. aries*	80	87	89	93	88	92	88	90	95	97	88	93	95	96	79	85	78	86	87	93
*E. caballus*	86	91	87	91	89	92	91	93	96	97	90	94	96	97	79	86	80	86	89	93
*S. scrofa*	86	91	88	92	89	92	91	93	95	97	89	94	95	96	80	86	79	86	87	91
*G. gallus*	74	81	74	84	60	71	79	86	91	95	74	85	73	83	57	70	-	-	75	84

I = identity percentage; S = similarity percentage. Alignments performed with BLAST tool at the NCBI database (https://blast.ncbi.nlm.nih.gov/Blast.cgi) accessed on 10 March 2023.

## Data Availability

Not applicable.
